# Open Letter

**Published:** 1890-09

**Authors:** W. L. Williams


### CORRESPONDENCE.

### OPEN LETTER.

W. H. Hoskins, D.V.S., *Secretary and Chairman Sub-Committee of Arrangements :*

Dear Sir : Your letter in the August Journal in reply to mine in the *Review*, contains more in quantity and quality of fraternal feeling of Eastern toward Western veterinarians than had previously gained publicity through the press. The West desired such declaration of fraternity, and it was largely exploratory, for the purpose of discovering if such feeling existed in the East, that my first letter was written, hence its main object has been realized in a very satisfactory manner. Incorporated in your letter, however, are some statements which deserve comment.

At the outset, in saying "It [the United States Veterinary Medical Association] has always in the past confined its programme to members of its organization, and only by special request given by unanimous vote, allowed outside papers to be read at its meetings," you would apparently have the readers of the Journal infer that my open letter was the result of wounded vanity at not being accorded a place on your programme. As this subject was not broached in my letter, it would appear incompetent in your reply, and since I have at no time, and in no manner, asked or desired the honor, nor asked any friend to speak for me in the matter, it would appear that you were laboring under misapprehension.

But were the suggestions as to my motive well placed, your statements as to usage seem at variance with the records of your society, since in your own city, Philadelphia, at your meeting of September 20th, 1886, a paper was read by an eminent gentleman who was not only not a member, but if your title, United States, means anything, he was not even *eligible* to membership, and was not on your honorary roll. It is abundantly safe to say he was *invited*, not *suffered*, to read his paper, after reverently begging the favor.

You grow phenomenally and unnecessarily eloquent over the fact that the published criticisms came not from a member but from one " who but a few years since had his name dropped for non-payment of initiation fee and dues."

You failed to add the very material fact that I did not apply for membership, as I was aware that all your meetings were held 1000 miles distant, out of financial reach of the young struggling Western veterinarian, and it was for the purpose of breaking down this barrier between you and the mass of Western veterinarians that we urged you to hold a Western meeting.

I can scarcely believe that your society makes a practice of electing veterinarians to membership without their having applied, and then pays you a salary as its authorized spokesman to belittle those who find it impracticable to accept the proffered honor.

To your claim that "no names of members from the West have been debarred [from programme], nor have any requests from Western members of the association been denied," we would say that Western veterinarians of good standing, whether members or not, are not likely to beg for a place, nor is it plain why you should expect them to do so, when by your own admissions and records you procure Eastern papers from members and non-members, through the active solicitation of your committee, while among the thousand Western veterinarians, with one known exception, you solicited no papers until after my open letter, and accorded them very scant attention, not even deigning to answer an urgent business letter from your fellow officer, State Secretary Butler, of Iowa, who, galled and disheartened by your silence, was finally driven to withdraw his paper, partially promised to your president.

It has become quite evident that the unpleasant tangle in which we became involved was due in the main, if not wholly, to a very natural error ; you broke away from a time-honored precedent to hold a meeting in the West, of an association which by holding its meetings in the East, has remained essentially Eastern, and after deviating from that precedent as to location, you overlooked the advisability of departing from custom in other matters as well, thus threatening the desired harmony between programme and place.

Whatever may be said of its drawbacks, public agitation of the subject has brought forth such frank and candid expressions of fraternal feeling toward Western veterinarians that it is very evident that your actions and utterances of an earlier date failed to faithfully portray the prevailing sentiments of the body of your society or even of your own personal feelings, after more careful reflection and observation under the new conditions and from the new point of view, brought about by change of location.

It now remains only for Western veterinarians to attend the Chicago meeting as largely as possible ; to enter fully and heartily into the enjoyment of your very excellent programme, the worth of which is fully assured by the names of Drs. Salmon, Huidekoper and Liautard ; to mingle cautiously and courteously in the business and social life of the society, and above all to demonstrate beyond doubt our hearty fraternal feeling toward Eastern veterinarians, becoming thoroughly amalgamated with them in a truly grand national organization, and extending to them so cordial a welcome that not only will they rejoice for having come among us but will gladly look forward to an early return to the West.

The meeting will prove one of unusual importance to the profession at large, offering, as it does by far the best opportunity in its history for the United States Veterinary Medical Association to become, in the highest sense, the representative national veterinary organization of the United States, and to this end all veterinarians from every section, who can possibly do so, should attend the meeting, and laying aside all sectional or personal feelings, unite in one whole, with no East, West, North or South, and with but one object in view—the elevation of our profession.

With the sincere wish that the Chicago meeting may prove of very great value to the society and profession, and heartily assuring that what has been said and done, however erratic or misguided, has been inspired only by a desire for that justice and respect of which we are now assured, to the large section of the profession of which I am a member. I remain,

Yours truly,

W. L. Williams.

